# A simple criterion to design optimal non-pharmaceutical interventions for mitigating epidemic outbreaks

**DOI:** 10.1098/rsif.2020.0803

**Published:** 2021-05-12

**Authors:** Marco Tulio Angulo, Fernando Castaños, Rodrigo Moreno-Morton, Jorge X. Velasco-Hernández, Jaime A. Moreno

**Affiliations:** ^1^ CONACyT - Institute of Mathematics, Universidad Nacional Autónoma de México, Juriquilla 76230, México; ^2^ Institute of Mathematics, Universidad Nacional Autónoma de México, Juriquilla 76230, México; ^3^ Department of Automatic Control, Cinvestav-IPN, Ciudad de México 07360, México; ^4^ Faculty of Sciences, Universidad Nacional Autónoma de México, Ciudad de México 04510, México; ^5^ Institute of Engineering, Universidad Nacional Autónoma de México, Ciudad de México 04510, México

**Keywords:** epidemic outbreak, non-pharmaceutical interventions, COVID-19, optimal control

## Abstract

For mitigating the COVID-19 pandemic, much emphasis is made on implementing non-pharmaceutical interventions to keep the reproduction number below one. However, using that objective ignores that some of these interventions, like bans of public events or lockdowns, must be transitory and as short as possible because of their significant economic and societal costs. Here, we derive a simple and mathematically rigorous criterion for designing optimal transitory non-pharmaceutical interventions for mitigating epidemic outbreaks. We find that reducing the reproduction number below one is sufficient but not necessary. Instead, our criterion prescribes the required reduction in the reproduction number according to the desired maximum of disease prevalence and the maximum decrease of disease transmission that the interventions can achieve. We study the implications of our theoretical results for designing non-pharmaceutical interventions in 16 cities and regions during the COVID-19 pandemic. In particular, we estimate the minimal reduction of each region’s contact rate necessary to control the epidemic optimally. Our results contribute to establishing a rigorous methodology to design optimal non-pharmaceutical intervention policies for mitigating epidemic outbreaks.

## 1. Introduction

Since the seminal work of May & Anderson [1], the design of interventions to *eradicate* infectious diseases has the objective of achieving a basic (*R*_0_) or effective reproduction number below one [2,3]. The underlying assumption here is that it is possible to maintain interventions for long periods, such as long-term vaccination programmes. During the COVID-19 pandemic, this same objective is guiding the design of non-pharmaceutical interventions (NPIs) [4]. However, maintaining NPIs like bans of public events or lockdowns for long periods of time is infeasible because of their substantial economic and societal costs [5,6]. Actually, instead of aiming for eradication, NPIs aim to *mitigate* the economic and social costs of an epidemic outbreak [7]. Nevertheless, we still lack simple guidelines to design NPIs for mitigating epidemic outbreaks, analogous to the *R*_0_ < 1 condition for eradication.

Here, we use the classic Susceptible–Infected–Removed (SIR) epidemiological model to fully characterize the design of NPIs for mitigating epidemic outbreaks. With this aim, we consider that NPIs should achieve an optimal tradeoff between two objectives [8]. First, optimal NPIs must minimize the period in which they need to be applied, consequently minimizing their associated economic and societal costs. Second, optimal NPIs must guarantee that the disease prevalence does not exceed a specified maximum level, which for example can represent health services’ capacity for that particular disease outbreak [9]. We obtain a full analytical characterization of such optimal NPIs for mitigating epidemic outbreaks, specifying the optimal intervention at each state that the epidemic can be. This characterization yields the necessary and sufficient criterion for the existence of optimal NPIs for mitigation, analogous to the *R*_0_ < 1 condition for eradication. We find that reducing the reproduction number below one is sufficient but not necessary for their existence. Instead, for mitigation, we show that the desired maximum disease prevalence determines the necessary reduction in the reproduction number. The consequence of not reducing the reproduction number below one is that interventions must start before the disease prevalence reaches the specified maximum level. We also demonstrate numerically that the derived optimal NPIs for mitigation are robust to uncertainties in the model parameters and unmodelled epidemic dynamics (e.g. undetected infections). Finally, we explore the implications of our theoretical result by analysing the response of 16 cities and regions across the globe to the COVID-19 pandemic, finding that most regions achieved a larger-than-necessary reduction in transmission. Our results contribute to designing NPIs to optimally and robustly mitigate epidemic outbreaks.

## 2. Characterizing optimal non-pharmaceutical interventions

### 2.1. Optimal epidemic mitigation using non-pharmaceutical interventions

**Figure 1.**
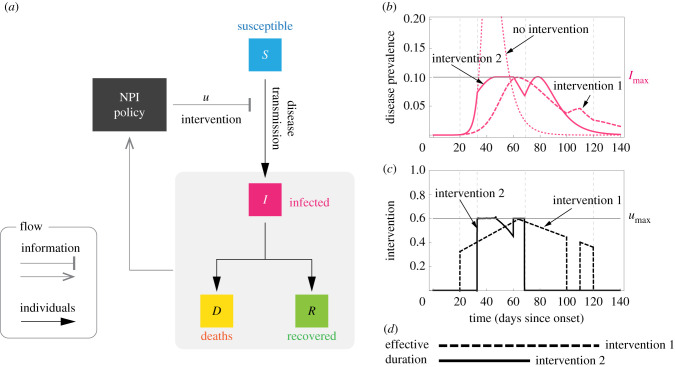
Optimal non-pharmaceutical interventions. (*a*) Susceptible–Infected–Removed (SIR) model with non-pharmaceutical interventions (NPIs) reducing disease transmission. For the optimal NPI design problem, the objective is to design the intervention u*(*t*) with minimal effective duration such that u*(*t*) ∈ [0, *u*_max_] and *I*(*t*) ≤ *I*_max_ for all *t* ≥ 0. (*b*,*c*) The response of the SIR model for two interventions (parameters are *β* = 0.52, *γ* = 1/7, *I*_0_ = 8.855 × 10^−7^ and *S*_0_ = 1 − *I*_0_). Both interventions 1 and 2 satisfy *u*(*t*) ≤ *u*_max_ and guarantee that *I*(*t*) ≤ *I*_max_. Actually, intervention 2 is the optimal one derived using our analysis: it is the intervention with minimal effective duration satisfying *I*(*t*) ≤ *I*_max_. (*d*) The effective duration of an intervention measures the interval between the start of the outbreak and the last time that a non-zero intervention is applied. In this example, the effective duration of intervention 1 is 120 days, while the effective duration of intervention 2 is 69 days.

Our objective is to characterize the reduction in the disease transmission that is optimal for each *state* in which the epidemic outbreak can be. For this, we leverage on the mathematical tractability of the SIR model [10], where the state can be characterized by the pair $(S,I)\in {\left[0,1\right]}^{2}$. Here, *S* is the proportion of the population that is susceptible to the disease, and *I* is the disease prevalence (i.e. the proportion of the population that is infected); see figure 1*a*. We discuss later other more detailed epidemic models. The epidemic state changes with time *t* as the disease is transmitted, producing the trajectory (*S*(*t*), *I*(*t*)) for *t* ≥ 0. For epidemic *mitigation*, we consider that the goal is keeping the disease prevalence below a specified level *I*_max_ ∈ (0, 1]. A main factor determining this constant is health services’ capacity in the sense that a prevalence above *I*_max_ causes higher mortality due to hospital saturation [9]. In general, the selection of *I*_max_ could depend on other social and economic factors of the specific population where the outbreak occurs. To keep *I*(*t*) ≤ *I*_max_, we assume we can apply one or several NPIs that reduce disease transmission by the factor (1 − *u*), for some *u* ∈ [0, 1]; see figure 1*a*. The NPIs achieve no reduction when *u* = 0, and they completely stop transmission when *u* = 1. Different NPIs correspond to particular values of *u*. For instance, a study of NPIs during the COVID-19 pandemic [11] found that closing most non-essential business corresponds to *u* ≈ 0.25, closing schools and universities to *u* ≈ 0.37, and limiting gatherings to at most 10 people to *u* ≈ 0.42. In practice, it can be unfeasible to fully stop the disease transmission (i.e. *u* < 1) because of inherent challenges like asymptomatic transmission [12], or because of the necessity of maintaining a working economy [13]. Therefore, we upper-bound the reduction by *u*_max_ ∈ (0, 1). We say that *u* is *admissible* if *u* ∈ [0, *u*_max_].

Different admissible NPIs can keep the disease prevalence below *I*_max_. For instance, ‘intervention 1’ in the example of figure 1*b*,*c* keeps this restriction and it has an ‘effective duration’ of 120 days. Here, the *effective duration* of an intervention is the time interval between the start of the outbreak and the last time that a non-zero intervention is applied (figure 1*d*). ‘Intervention 2’ of figure 1*b*,*c* also keeps the restriction *I*(*t*) ≤ *I*_max_, but its effective duration is only 69 days. To design the *optimal* NPI for mitigating epidemic outbreaks, we ask for the intervention with minimal effective duration. Specifically, we ask for the admissible intervention u*(*S*(*t*), *I*(*t*)) required *now* (i.e. at the current state) such that: (1) it minimizes the effective duration of the intervention and (2) it ensures that the prevalence can be maintained below *I*_max_ for *all future* time by using some admissible intervention. If the optimal NPI problem has a solution u*, then u*(*S*, *I*) characterizes the optimal reduction in the disease transmission that the NPIs should achieve if the epidemic state is (*S*, *I*). In particular, u* gives the optimal way to start and stop the NPIs.

### 2.2. Optimal non-pharmaceutical interventions for mitigation exist without reducing the reproduction number below one

**Figure 2.**
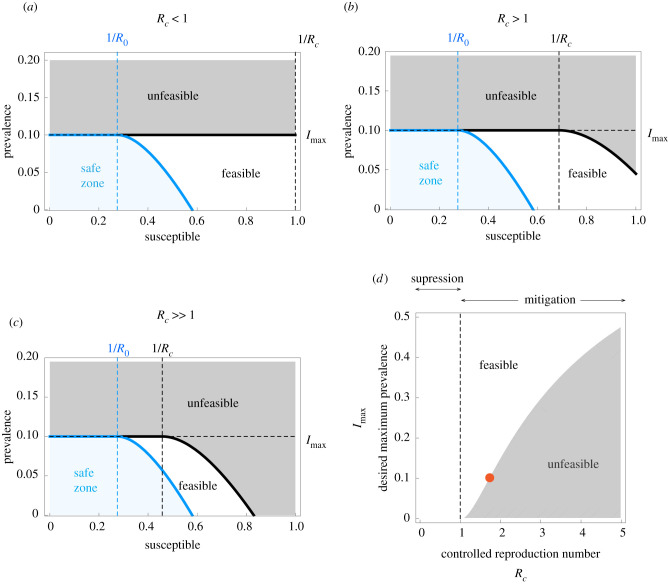
Existence of non-pharmaceutical interventions in the Susceptible–Infected–Removed model. Parameters are *γ* = 1/7, *β* = 0.52 (i.e. *R*_0_ = 3.64) and *I*_max_ = 0.1. The safe zone (in blue) consists of all states that do not exceed *I*_max_ without interventions. This zone is characterized by the inequality $I\leq \Phi_{R_{0}}(S)$. The plane is further divided into feasible states that can reach the safe zone without exceeding *I*_max_ (white), and unfeasible states that cannot (grey). Feasible and unfeasible states are separated by the separating curve $\Phi_{R_{c}}(S)$ (black line). (*a*) For ‘strong’ interventions with *u*_max_ = 0.8, the controlled reproduction number is *R*_*c*_ = (1 − *u*_max_)*R*_0_ = 0.728 < 1. Here, the separating curve is the straight line *I*_max_, implying that all states below *I*_max_ are feasible. Note this case corresponds to eradication. (*b*) For ‘intermediate’ interventions with *u*_max_ = 0.6, the controlled reproduction number is *R*_*c*_ = (1 − *u*_max_)*R*_0_ = 1.456 > 1. Here, the separating curve $\Phi_{R_{c}}(S)$ is nonlinear, and some states below *I*_max_ are unfeasible. (*c*) For ‘weak’ interventions with *u*_max_ = 0.4 we obtain *R*_*c*_ = 2.184 > 1. In this case, states with *S*(0) ≈ 1 are unfeasible. (*d*) For *S*(0) → 1, our design criterion for NPIs prescribes the values of *R*_*c*_ that a given *I*_max_ can manage.

Our first main result is a complete analytical characterization of the optimal NPIs for outbreak mitigation in the SIR model (see box 1 for a summary and electronic supplementary material, note S1, for details). To understand how these optimal NPIs work, note that the SIR model predicts a *safe zone* of states (*S*, *I*) where, without any further interventions, the disease prevalence will not exceed *I*_max_ (blue zone in figure 2*a*–*c*). The safe zone is characterized by the inequality $I\leq \Phi_{R_{0}}(S)$, where *R*_0_ is the *basic reproduction number* of the outbreak in the population, and the function $\Phi_{R}$ is defined in equation (2.1) of box 1. The goal of the optimal NPIs is thus to reach this safe zone as fast as possible without violating the restriction *I*(*t*) ≤ *I*_max_. The ability to achieve this goal depends on the epidemic state. That is, we can partition the plane (*S*, *I*) in two regions: those states from which it is possible to reach the safe zone without exceeding *I*_max_ (*feasible* states), and those where it is impossible (*unfeasible* states). We find these two regions are characterized by the separating curve $\Phi_{R_{c}}(S)$, where we call *R*_*c*_ := (1 − *u*_max_)*R*_0_ the *controlled reproduction number* (figure 2*a*–*c*). Note that *R*_*c*_ describes the maximum reduction in the basic reproduction number that (constant) admissible interventions can achieve. Therefore, *R*_*c*_ < 1 is the necessary and sufficient condition that a constant and permanent admissible intervention (i.e. *u*(*t*) ≡ const. for all *t* ≥ 0) needs to satisfy to *eradicate* a disease outbreak in the SIR model. However, for outbreak mitigation, our analysis shows that feasible states exists without achieving disease eradication (white regions in figure 2*b*,*c*). This result is important because it proves that optimal NPIs for epidemic mitigation do not require reducing the basic reproduction number below one (i.e. without achieving *R*_*c*_ < 1).

Box 1. **Optimal NPIs for the Susceptible–Infected–Removed (SIR) model.**

The SIR model with interventions *u*(*t*) ∈ [0, *u*_max_] reducing disease transmission takes the form

$$\frac{dS}{dt}=-(1-u)\beta \;SI,\;\frac{dI}{dt}=(1-u)\beta \;SI-\gamma I.$$

Here, *S*(*t*) and *I*(*t*) are the proportion of the population that is susceptible or infected at time *t* ≥ 0, respectively. We denote by (*S*_0_, *I*_0_) the initial state at *t* = 0. The parameters of the SIR model are the (effective) *contact rate* *β* ≥ 0, and the mean *residence time* of infected individuals *γ* ≥ 0 (in units of day^−1^). By assuming *S*_0_ ≈ 1, these two parameters yield the *basic reproduction number* *R*_0_ = *β*/*γ*.

We are interested in reaching the *safe zone*

$$S=\{(S,I)|I\leq \Phi_{R_{0}}(S)\},$$

where

2.1 $$\Phi_{R}(S)=\{\begin{array}{ll} I_{\max} & \mathrm{if}\;S\leq R^{-1},\\ I_{\max}+R^{-1}[\log(RS)+1-RS] & \mathrm{otherwise}.\; \end{array}$$

The safe zone is the largest set with the following property: if, for any given time *t*_1_, the state (*S*_1_, *I*_1_) belongs to S, we can set *u* = 0 henceforth and still have *I*(*t*) ≤ *I*_max_ for all *t* ≥ *t*_1_. That is, when S is reached, we can terminate the intervention with the assurance that a possible rebound in the disease prevalence will not exceed *I*_max_.

Our goal is to steer an arbitrary initial state (*S*_0_, *I*_0_) to the safe zone S in minimal time without violating the constraint *I*(*t*) ≤ *I*_max_. We say that an intervention achieving this goal is an *optimal intervention*.

In electronic supplementary material, note S1, we prove that the existence of an optimal intervention is characterized by the *separating curve* $\Phi_{R_{c}}$ as follows:

1. An optimal intervention exists if and only if the initial state (*S*_0_, *I*_0_) lies below this separating curve (i.e. $I_{0}\leq \Phi_{R_{c}}(S_{0})$).

 Above, *R*_*c*_ : = (1 − *u*_max_)*R*_0_ is the *controlled reproduction number*. Moreover:

2. If it exists, the optimal intervention u* at the state (*S*, *I*) is

2.2 $$u^{*}(S,I)=\{\begin{array}{ll} 0 & \mathrm{if}\;(S,I)\in S\cup W\\ 1-\frac{1}{R_{c}S} & \mathrm{if}\;I=\Phi_{R_{c}}(S)\;\mathrm{and}S^{*}<S<R_{c}^{-1}\\ u_{\max} & \mathrm{otherwise} \end{array}$$

with

$$W=\{(S,I)|I<\Phi_{R_{c}}(S),\;S>\Psi (I)\}.$$

 Above, the curve S=Ψ(I) is defined in electronic supplementary material, note S1, while S* denotes the intersection of S=Ψ(I) and $I=\Phi_{R_{c}}(S)$.

Code to calculate the optimal interventions is provided as electronic supplementary material.

### 2.3. A design criterion for optimal non-pharmaceutical interventions

We demonstrated above that optimal NPIs for outbreak mitigation exist even when *R*_*c*_ > 1. However, how large can *R*_*c*_ be before NPIs keeping *I*(*t*) ≤ *I*_max_ do not exist? When *S*(0) → 1, our characterization shows that such NPIs exists if and only if

2.3 $$R_{c}\leq 1,\;\text{or}\;I_{\max}+\frac{1}{R_{c}}\ln R_{c}-\left(1-\frac{1}{R_{c}}\right)\geq 0.$$

The above inequality is our second main result, connecting the specified maximum disease prevalence *I*_max_ with the outbreak’s controlled reproduction number *R*_*c*_ = (1 − *u*_max_)*R*_0_ (electronic supplementary material, note S2). The inequality (2.3) governs the existence of NPIs for mitigating epidemic outbreaks, in analogy to how the condition *R*_*c*_ < 1 works for disease eradication. Note that *R*_*c*_ < 1 is a sufficient condition for the existence for NPIs, but the inequality (2.3) shows that this condition is far from necessary. If *I*_max_ > 0, there exists *R*_*c*_ > 1 for which NPIs exist (figure 2*d*). Note also that the maximum feasible *R*_*c*_ increases with *I*_max_.

We can use (2.3) to design NPIs for outbreak mitigation as follows. Consider an infectious disease outbreak with a given *R*_0_ and that the specified maximum prevalence is *I*_max_. Then, the inequality (2.3) gives the criterion to design NPIs by providing the range of disease transmission reduction *u*_max_ that the NPIs should attain. In particular, it provides the minimal reduction $u_{\max}^{*}$ in the disease transmission required for the existence of NPIs. For example, if *I*_max_ = 0.1 then $R_{c}^{*}=1.71$ is the maximum admissible controlled reproduction number (orange point in figure 2*d*). Therefore, if an outbreak in the population has *R*_0_ = 3, then the minimal required reduction is $u_{\max}^{*}=0.43$ because $(1-u_{\max}^{*})R_{0}=R_{c}^{*}$.

### 2.4. Optimal non-pharmaceutical interventions for outbreak mitigation are simple

**Figure 3.**
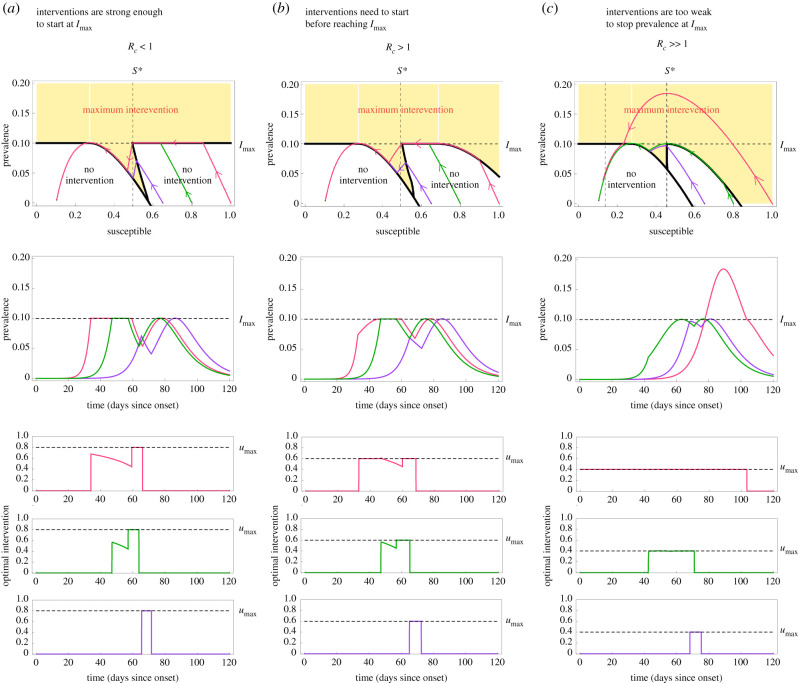
Optimal non-pharmaceutical interventions in the Susceptible–Infected–Removed model. For all panels, the parameters of the SIR model are *γ* = 1/7, *β* = 0.52 (i.e. *R*_0_ = 3.64) and *I*_max_ = 0.1. We consider a population of *N* = 8.855 × 10^6^ individuals (like in Mexico City) and *I*_0_ = 1/*N*. Panels shows trajectories for three initial proportions of the susceptible population: large *S*_0_ = 1 − *I*_0_ ≈ 1 (pink), medium *S*_0_ = 0.8 (green) and small *S*_0_ = 0.65 (purple). (*a*) For *u*_max_ = 0.8, we have *R*_*c*_ = (1 − *u*_max_)*R*_0_ = 0.728 ≤ 1. In this case, the optimal intervention starts when the disease prevalence reaches *I*_max_. Afterwards, the intervention decreases in a hyperbolic arc until reaching the point *S* = S*. At that time, the intervention becomes maximum in the ‘final push’ to reach the safe zone. (*b*) For *u*_max_ = 0.58, the controlled reproduction number is *R*_*c*_ = (1 − *u*_max_)*R*_0_ = 1.52 > 1. Here, $\Phi_{R_{c}}(1)>0$, implying that the epidemic still can be mitigated for initial states with *S*_0_ ≈ 1 and *I*_0_ ≈ 0 (pink trajectory). In this case, the optimal intervention starts when the initial condition hits the separating curve below *I*_max_ at *t* = 35. At that instant, the intervention starts with the maximum value *u*_max_, and continues in that form until the trajectory reaches *I*_max_. (*c*) Choosing *u*_max_ = 0.4 yields *R*_*c*_ = 2.184 > 1. In this case, the optimal intervention problem does not have a solution for all initial states *S*_0_ > 0.85. This is illustrated by pink trajectory: even when applying the maximum intervention from the start, *I*(*t*) will grow beyond *I*_max_.

For any epidemic state, the optimal transmission reduction takes a simple form which can be described by colouring the (*S*, *I*) plane; see top row of figure 3. Here, for all states in the white region the optimal intervention is no intervention; for all states in the yellow region the optimal intervention is u*(*S*, *I*) = *u*_max_. There are regions (specifically lines) where the optimal intervention switches frequently between u* = 0 and u* = *u*_max_ producing a so-called ‘singular arc’ that slides along the two regions, leading to an ‘average’ intervention u* ∈ [0, *u*_max_]. In general, we find that the optimal NPIs have four phases: a first one where no intervention is needed, a second phase where interventions start with maximum strength, a third phase of gradual decrease of interventions, and a ‘final push’ where the maximum interventions are re-applied for a short period to reach the safe zone faster.

We illustrate the above behaviour in three qualitatively different cases. The first case is when the optimal intervention starts just when the disease prevalence reaches *I*_max_ (figure 3*a*). This case occurs when the interventions are strong enough to stop the rise in prevalence at *I*_max_ regardless of the remaining fraction the population that is still susceptible to the disease. Our analysis shows that this case occurs if and only if *u*_max_ is large enough to render *R*_*c*_ = (1 − *u*_max_)*R*_0_ ≤ 1. When the initial susceptible population is close to 1 (pink trajectory in figure 3*a*), the optimal intervention first waits until the disease prevalence reaches *I*_max_. At that time, the optimal NPI stops the disease prevalence exactly at *I*_max_, and then it gradually decreases its magnitude to ensure that the disease prevalence slides along *I*_max_ as the susceptible population decreases. When the susceptible population reaches the threshold S*, the optimal intervention is again the maximum one (figure 3*a*). This ‘final push’ allows reaching the safe zone faster, releasing the interventions sooner. The middle and bottom panels of figure 3*a* show the resulting disease prevalence and optimal interventions as a function of time. Note that a smaller initial susceptible population yields other trajectories (green and purple in figure 3*a*).

The second case is when an ‘early’ intervention is necessary before the disease prevalence reaches *I*_max_ (figure 3*b*). This case happens when the admissible reduction in the contact rate cannot immediately stop the disease prevalence at *I*_max_ if the susceptible population is large at that time. We find this case occurs if and only if *u*_max_ is small in the sense that *R*_*c*_ = (1 − *u*_max_)*R*_0_ > 1. Here, a trajectory may hit the yellow region before reaching *I*_max_ (pink trajectory in figure 3*b*). When that happens, the optimal intervention starts with the maximum reduction u* = *u*_max_. Then it maintains this maximum reduction to ‘slide’ the trajectory between the yellow and white regions. Once the trajectory reaches *I*_max_, the magnitude of the optimal intervention decreases to slide the trajectory along *I*_max_. Again, the final push occurs when the susceptible population reaches the point S*.

The third case is when the initial state (*S*_0_, *I*_0_) lies in the unfeasible region (figure 3*c*). This case occurs when *u*_max_ is so small that, even if the maximum admissible intervention *u* = *u*_max_ is applied from the start of the outbreak, the disease prevalence will exceed *I*_max_ (pink trajectory in figure 3*c*). In this case, the optimal intervention problem is unfeasible because it is impossible to achieve *I*(*t*) ≤ *I*_max_. However, note that using u* = *u*_max_ yields the smallest prevalence peak. Other trajectories that start with a smaller proportion of susceptible individuals remain feasible (green and purple in figure 3*c*). In particular, note that the threshold S* decreases as *R*_*c*_ increases.

### 2.5. Optimal non-pharmaceutical interventions for outbreak mitigation are robust

To evaluate the optimal NPIs for outbreak mitigation in more realistic scenarios, we numerically analysed their performance in three epidemic models with uncertain epidemic parameters and more detailed epidemic dynamics (see details in electronic supplementary material, note S3). In all cases, we consider that the basic reproduction number has been estimated as ${\hat{R}}_{0}$ using an SIR model, and that the optimal NPIs for mitigation are designed using this estimate. Then, these optimal NPIs are applied to an outbreak with possibly different epidemic dynamics and possibly different *R*_0_. Note that estimation errors in *R*_0_ will affect the correct start of the NPIs and the ‘final push’ for reaching the safe zone.

**Figure 4.**
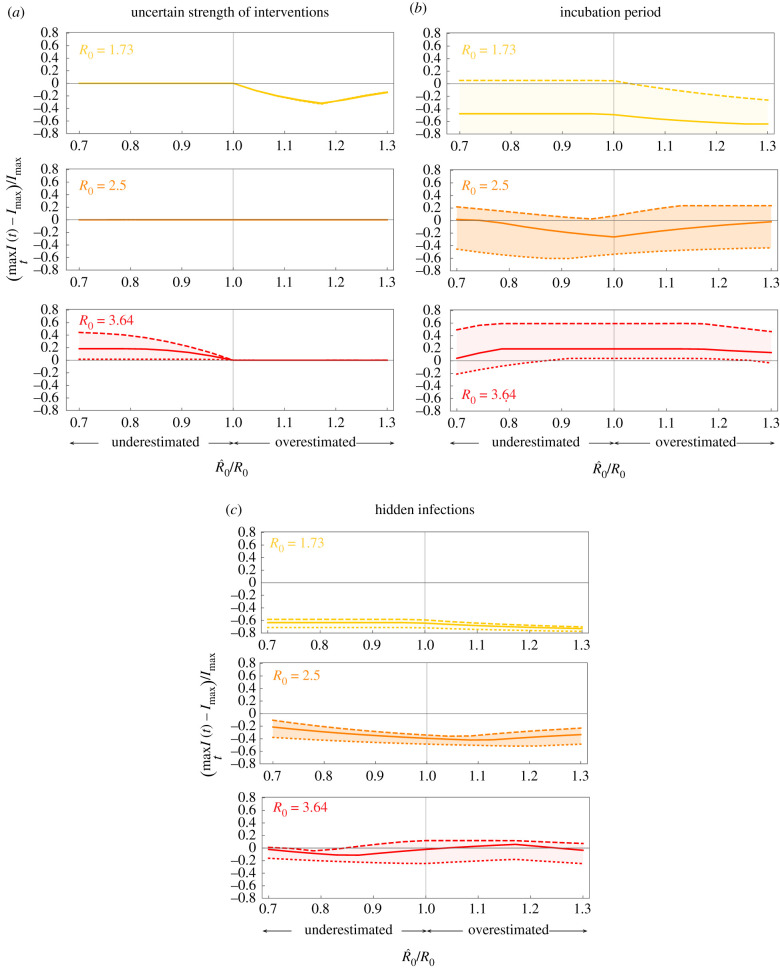
Optimal non-pharmaceutical interventions are robust. For all panels, the estimated parameters used for constructing the optimal NPIs are $\hat{\gamma }=1/7$, $\hat{\beta }=0.52$, *I*_max_ = 0.1, *u*_max_ = 0.6. We consider a population of *N* = 8.855 × 10^6^ as in Mexico City, and the initial conditions *I*(0) = 1/*N* and *S*(0) = 1 − 1/*N*. If the models contain other state variables, they were initialized at zero. The optimal NPIs are constructed assuming ${\hat{R}}_{0}=\hat{\beta }/\hat{\gamma }$, while the actual epidemic dynamics has a possibly different *R*_0_ = *β*/*γ*. Panels show results for outbreaks with three values of *R*_0_: low (yellow), medium (orange) and large (red). (*a*) SIR model where the reduction in the disease transmission by the NPIs is uncertain. We model this case replacing *u* by *ku* in the model equations. Panel shows the results for *k* = 1.1 (dotted), *k* = 1 (solid) and *k* = 0.9 (dashed). (*b*) SEIR model where exposed individuals do not transmit the infection, with *λ* > 0 the incubation period. Panel shows the results for *λ* = 1/5 (dotted), *λ* = 1/7 (solid) and *λ* = 1/11 (dashed). (*c*) SEIIR model with *λ* = 1/7 and two classes of infected individuals (symptomatic and asymptomatic). Here, *p* ∈ [0, 1] is the proportion of exposed individuals that become asymptomatic. The vertical axis denotes the disease prevalence for symptomatic individuals. The panel shows the results for *p* = 0.55 (dotted), *p* = 0.7 (solid) and *p* = 0.8 (dashed).

In the first scenario, we consider an outbreak with SIR dynamics where the strength of the NPIs is uncertain. We model this uncertainty replacing *u* by *ku* in the model equations, where *k* ∈ (0, 1). Then, for example, *k* = 0.9 (resp. *k* = 1.1) represents a 10% underestimation (resp. overestimation) of the NPI strength. Across outbreaks with different *R*_0_’s and an uncertainty of 10% in the intervention’s strength, we find that the disease prevalence is maintained below *I*_max_ as long as *R*_0_ is not underestimated (figure 4*a*). In the second scenario, we consider an SEIR outbreak with an incubation period for the disease. For an incubation period of 7 days, as typical for a COVID-19 infection, the optimal NPIs maintain the disease prevalence below *I*_max_ if *R*_0_ < 2.5 and its value is estimated with an error of below 30% (solid yellow and orange in figure 4*b*). For larger *R*_0_ or a larger incubation period, the disease prevalence may exceed *I*_max_ (red in figure 4*b*).

For the final scenario, we consider an SEIIR model with an incubation period of 7 days and with a fraction *p* ∈ [0, 1] of infected individuals that are asymptomatic and thus remain hidden to the epidemic surveillance system. The goal is to maintain the prevalence of symptomatic individuals below *I*_max_, without knowing the fraction of asymptomatic individuals. This situation occurs during the COVID-19 pandemic, where between *p* = 0.55 and *p* = 0.8 of infections are asymptomatic [14]. For *p* < 0.7 and *R*_0_ < 3.64, the optimal NPIs maintain the disease prevalence of symptomatic individuals below or very close to *I*_max_ if the estimation error for *R*_0_ is below 30% (dotted and solid lines in figure 4*c*). An outbreak with low *R*_0_ produces a maximum disease prevalence of symptomatic individuals below *I*_max_, which may result in a larger effective duration of the interventions. Overall, these numerical results show that the optimal NPIs are robust against a wide range of parameter uncertainty and unmodelled dynamics, provided that the estimation error in the outbreak’s basic reproduction number does not exceed 30%.

## 3. Designing optimal non-pharmaceutical interventions for mitigating the COVID-19 pandemic

**Figure 5.**
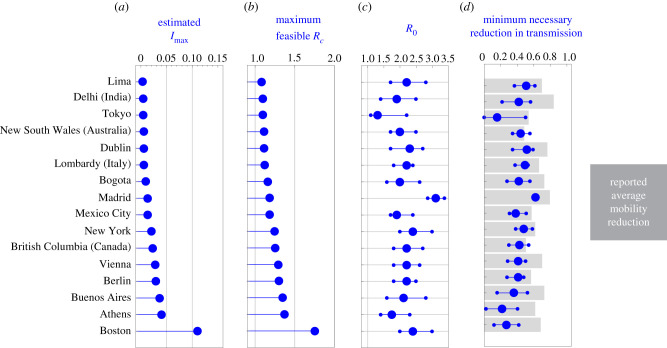
Minimum necessary reduction in disease transmission for NPIs in the COVID-19 pandemic. (*a*) Calculated *I*_max_ according to the proportion of available intensive care beds in each region or city and the estimated fraction of infected individuals requiring intensive care. (*b*) Maximum controlled reproduction number *R*_*c*_ that each region or city can handle according to its *I*_max_. Larger *I*_max_ allows a larger *R*_*c*_. (*c*) Basic reproduction number *R*_0_ per region or city before interventions started. Median (blue big dot), and 95% confidence interval (smaller dots) are shown. (*d*) Minimum *u*_max_ necessary for feasibility for each region or city (blue) according to the *R*_0_ of (*c*). Grey bars denote the reported average mobility reduction in each region between 19 March and 30 April 2020.

To explore the implications of our simple criterion for designing NPIs for outbreak mitigation, we analysed how 16 cities and regions implemented NPIs during the COVID-19 pandemic. For each region or city, we constructed *I*_max_ using the number of available intensive care beds during the first months of the pandemic, considering that a fraction of the infected individuals will require them (electronic supplementary material, note S4). The values for *I*_max_ that we obtain range from 2.87 × 10^−3^ for Lima (Peru) to 109.78 × 10^−3^ for Boston (USA), reflecting the large heterogeneity of the available health services across the globe (figure 5*a*). With this information, we calculated the maximum feasible $R_{c}^{*}$ for each region using our design criterion of inequality (2.3). Since $R_{c}^{*}$ is a monotone function of *I*_max_, we find that $R_{c}^{*}$ follows the same trend as *I*_max_ (figure 5*b*). The smallest $R_{c}^{*}=1.08$ occurs for Lima and the largest $R_{c}^{*}=1.75$ for Boston. Note that in both cases $R_{c}^{*}>1$. This result implies that, for the *R*_0_ of a region’s disease outbreak, a successful mitigation of the outbreak requires NPI policies that achieve at least a reduction $u_{\max}^{*}$ such that $(1-u_{\max}^{*})R_{0}\leq R_{c}^{*}$.

Next, we investigated the *minimal* reduction $u_{\max}^{*}$ in transmission required to achieve those upper bounds for the COVID-19 pandemic. For this, we first collected information for the *R*_0_ in each region calculated at the start of the pandemic and when the NPIs were inactive (electronic supplementary material, note S3). We find a median nominal *R*_0_ of 2.2, with Tokyo having the smallest one (*R*_0_ = 1.3) and Madrid having the largest one (*R*_0_ = 3.11); see figure 5*c*. From these values of *R*_0_, we calculated the minimal required reduction $u_{\max}^{*}$ per region or city (blue in figure 5*d*). For the nominal *R*_0_’s per region or city, we find that a median reduction of $u_{\max}^{*}$ of 0.42 is necessary. However, this minimal necessary reduction is heterogeneous across regions. For example, Tokyo just requires $u_{\max}^{*}=0.15$ while Madrid requires $u_{\max}^{*}=0.61$. These two cities have the smallest and largest *R*_0_, respectively. If two cities have similar *R*_0_, then the city with large *I*_max_ ends up requiring a smaller $u_{\max}^{*}$ (e.g. Boston with $u_{\max}^{*}=0.26$ and Lima with $u_{\max}^{*}=0.50$).

To evaluate the feasibility of achieving the minimal reduction predicted by our analysis, we collected data for the average mobility reduction in each region during the NPIs (grey in figure 5*d*; electronic supplementary material, note S4). Considering this average mobility reduction as a proxy for the reduction in disease transmission, we find that all regions achieved a greater than necessary reduction. For example, Delhi attained a mobility reduction of 0.84, while the minimal necessary reduction in transmission according to our analysis is $u_{\max}^{*}=0.42$. Other regions are in the boundary. For example, New South Wales attained a mobility reduction of 0.48, while the minimal necessary reduction in transmission was $u_{\max}^{*}=0.44$. Overall, across regions, we find a median excess of 0.22 in the reduction of mobility compared to the minimal reduction in transmission $u_{\max}^{*}$ predicted by our analysis.

## 4. Discussion

Our results provide a complete analytical characterization of the optimal NPIs for mitigating epidemic outbreaks in the SIR model. We also show that these optimal NPIs are robust as they can ‘work’ in epidemic models with more complicated dynamics. The SIR model is a minimal strategic model of the general population dynamics of a disease. Although this model ignores critical epidemiological phenomena, using the SIR model allows us to leverage on its mathematical tractability to obtain a complete characterization of the optimal NPIs for outbreak mitigation. The feedback form u*(*S*, *I*) of the optimal intervention reflects such complete characterization, prescribing the optimal action to perform if the epidemic is at any state (*S*, *I*). This feedback strategy should be contrasted to most other studies applying optimal control to epidemic outbreaks, where the derived optimal intervention u*(*t*, *S*_0_, *I*_0_) is an open-loop function of time [15–18]. The open-loop intervention gives the optimal action at any time given a particular initial state (*S*_0_, *I*_0_). However, it does not tell us the optimal action if the epidemic is not in the exact state predicted by the model.

Understanding the optimal action to perform at any state has the crucial advantage of allowing us to apply this knowledge to any model, and to reality. Feedback can give control strategies the required robustness to work on real systems despite large uncertainties and unknown dynamics [19,20], and we numerically confirmed that our optimal NPIs for outbreak mitigation have such robustness. Indeed, other works have also found that interventions derived from the SIR model can work in detailed agent-based models of epidemic outbreaks [21]. Future work could analyse the robustness of optimal interventions when the epidemic state is not entirely known. This situation may happen when significant delays exist in reporting new infections, or when tests for identifying infected individuals are limited. For example, control-theoretical techniques like the construction of observers and predictors allow applying our optimal interventions when the only available information is the disease prevalence, and when this information is obtained with a significant delay [22].

Our framework could also guide the complete characterization of optimal NPIs for mitigating epidemic outbreaks using more detailed models or more detailed optimization objectives, but this is likely very challenging. Indeed, deriving such complete characterization for very detailed models can be unreasonable, considering the tradeoff between how detailed is a model and how much we can trust its predictions [23]. Note also that our approach could be applied to calculate the optimal NPIs in the presence of a constant vaccination rate by modifying the SIR model accordingly (e.g. [24]).

The optimal intervention resulting from our analysis can take a continuum of values that may be infeasible to implement in practice. We can use an averaging approach to circumvent this problem. Namely, consider a time window of *T* days (e.g. a week). Suppose that the average reduction prescribed by the optimal intervention over a certain window is ${\bar{u}}^{*}$. We can realize this reduction on average by combining $d=T{\bar{u}}^{*}/u_{\max}$ days of maximum reduction with (*T* − *d*) days without intervention. This approach yields an intervention similar to Karin *et al*. [25], with the difference that the periods of intervention and activity are optimally balanced. Quantifying the errors produced by such approximations, in particular over the singular arc [26], deserves further study.

Our criterion to design optimal NPIs for mitigating epidemic outbreaks is obtained by characterizing the necessary and sufficient conditions for the existence of solutions to an optimal control problem. Specifically, the low dimensionality of the SIR model allowed us to apply Green’s theorem to compare the cost of any two interventions analytically (electronic supplementary material, note S1.4). In this sense, the method we use to derive the optimal NPIs is closer to our previous work on optimal control for bioreactors [27]. In general, deriving such complete characterization of optimal control problems is challenging because it involves solving an infinite-dimensional optimization [28]. Indeed, computational methods cannot produce such a characterization [29], and established analytical methods like Pontryagin’s maximum principle only yield necessary conditions for optimality [28]. Several works have applied these and other optimal control methods to the SIR model (e.g. [30,31]). The COVID-19 pandemic has produced a surge in the development of numerical and analytical methods to design optimal NPIs minimizing diverse criteria, including the infection peak [32], number of infections [33,34] and economic costs [35,36].

We will inevitably face new epidemics where NPIs are the only option to control the outbreaks. Rather counterintuitively, we find that for ‘ending’ an epidemic outbreak as fast as possible using NPIs, it is not always optimal to apply the maximum intervention. This observation illustrates the need for developing a better scientific understanding that informs the design of optimal NPIs and planning the required health services capacity.

## Data accessibility

The data analysed are reported in electronic supplementary material, notes S3 and S4. Code supporting the results is archived in the Github repository: https://github.com/SyntheticDynamics/OptimalOutbreakMitigation.
